# Morphing a Stereogram into Hologram

**DOI:** 10.3390/jimaging6010001

**Published:** 2020-01-02

**Authors:** Enrique Canessa, Livio Tenze

**Affiliations:** Science Dissemination Unit (SDU), ICTP—International Centre for Theoretical Physics, 34151 Trieste, Italy

**Keywords:** multi-view display, light-field rendering, optical flow

## Abstract

We developed a method to transform stereoscopic two-dimensional (2D) images into holograms via unsupervised morphing deformations between left (L) and right (R) input images. By using robust DeepFlow and light-field rendering algorithms, we established correlations between a 2D scene and its three-dimensional (3D) display on a Looking Glass HoloPlay monitor. The possibility of applying this method, together with a lookup table for multi-view glasses-free 3D streaming with a stereo webcam, was also analyzed.

## 1. Introduction

Digital technologies to engage with three-dimensional (3D) objects and environments have been a burgeoning and popular subject in recent years. The possibility of having a 3D vision display is highly beneficial in a range of diverse innovative scientific, engineering, and entertainment applications, including medicine, astro stereo photography, robotic vision, intelligent transportation systems, and video games, among others [1]. For example, it has been recently shown that the advantages of 3D stereoscopic visualization over a conventional two-dimensional (2D) planar screen can shorten the dissection time of specialized surgery [2]. Although many advances have been achieved already, a key limitation for these technologies is the fact that, in some cases, it can produce contagious yawning [3]. It is also necessary to wear special goggles or headsets, causing motion sickness and limiting user time. Notwithstanding such constraints, such applications can still give science a new dimension allowing researchers to view and share 3D data [4]. The final goal, however, would be to obtain and visualize similar results without wearing any device.

Motivated by the study of Ryan Baumann on animating stereograms with optical flow morphing [5], in this paper, we made a first attempt to reconstruct reality in similar simple terms by means of multi-views. We developed a method to combine observations of 2D stereoscopic images with 3D interpretations of reality. We applied the unsupervised torch-warp optical flow algorithm [5,6] to animate stereo pairs and retrieve distinct 2D views from morphing deformations between left (L) and right (R) images.

Since all these sequential frames put together can give an acceptable illusion of depth and parallax in the horizontal direction [5], we placed them in a single standard Quilt collage [7]. We then converted the Quilt into a Native light-field image using open source SURFsara visualization python scripts [8]. This Quilt provides a good visualization of virtual 3D imagery of stereograms by a direct display of the light-field output images on the new class of standalone Looking Glass 3D monitors [9]. Multiple viewers can see the scene inside stereoscopic images without the need for glasses and from different angles. We discuss on the possibility of applying this method for producing multi-view 3D streaming with a stereo webcam and the adoption of a pixel-and device-based Lookup Table (LUT).

Our work is, in fact, a development work, implementing a tool using some initial established technologies, extended to Linux Ubuntu O.S and incorporating a new LUT. When the Quilt representation is created, we map each of those pixels in the destination multi-view image.

## 2. Materials and Methods

We used low-cost ELP-960P2CAM (V90 and LC1100) USB stereo webcams with no distortion dual lens and M12 mount synchronization to obtain the principal 2D stereoscopic images [10]. According to specifications, the two camera video—with low power consumption, 90 degree lens, standard electronic rolling shutter, and 1/3${}^{\prime \prime }$ CMOS OV9750 sensor for high quality image—can reach high frame rates in MJPEG compression format of 2560(H) × 960(V)p@60fps with a sensitivity 3.7V/lux-sec@550 nm. Its small size of 80 × 16.5 mm is useful for embedded applications. It supports Linux OS USB video class UVC with adjustable parameters, such as brightness, contrast, saturation, hue, sharpness, color balance, and exposure.

For the multiple angles visualization of the morphing of stereograms into hologram image files, generated in full color with the present algorithm, we used the glasses-free standard Looking Glass 8.9${}^{\prime \prime }$ (also known as HoloPlay) as an HDMI external monitor [10]. This HoloPlay device combines light-field and volumetric technologies within a single new type of display, which allows the display of a hologram of simultaneous 32 (or more) different views at 60 fps formed via a 8 × 4 (or 9 × 5) Quilt input. The technology used in this class of monitor is described in the U.S. Patent application number 2017-0078655: *“Printed Plane 3D Volumetric Display”*.

Besides the standard 64 bit Windows 10, the viewing under O.S. Linux Ubuntu 19.04 was also possible using a Notebook Aspire E 15, Inter Core i5, 64 bit, 8 GIB RAM, 1366 × 768p resolution with graphics card Nvidia GeoForce 820 M output 2560 × 1600p. The small Raspberry Pi 3 single-board computer device Model B+ 1.4 GHz 64-bit quad-core processor with extra power supply was also able to display our stereograms morphed into a multi-view 3D display.

The procedure adopted to get a stereogram is as follows: We first position the principal object in the scene, at least 1.6 m distant apart from the two lenses of the ELP stereo webcam. This distance enables us to avoid image distortions due to well-known technical limitations of stereoscopic webcams [11]. The dual lenses are aligned parallel toward the horizon, avoiding to tilt the ELP’s device and limiting the depth view due to the reduction of near convergence.

The most outstanding feature of the compact ELP synchronized stereo webcam we used is that the two cameras’ video frames are synchronous. This unique feature enables simulation of the manner in which human eyes observe “simultaneously” one scene from two different viewpoints [10]. This is ideal for binocular stereo vision development, like the one studied in this work. By one single shot, we retrieve a single image in high definition (HD) resolution, containing L and R views, and without the need for any extra, complex prior calibration of the stereo webcams as in the case of most compact industrial camera devices, producing and displaying two L and R images independently.

We take with the ELP a stereoscopic test picture using the *ffmpeg* command and cut the resulting single HD image of max. resolution of 1280 × 960p in two equal parts to generate the L and R images. We then resize each view to set initially the widths to 512 p and then crop their heights to 256 p (anywhere along its vertical axis starting from the same upper corner in both images). This image manipulation is carried out before the morphing rendering to speed up the computation using smaller images and avoid the case of an *"out-of-memory (RAM) condition before sgemm"* in the deep convolution matching [6] when trying to match the full HD images.

**Figure 1:**
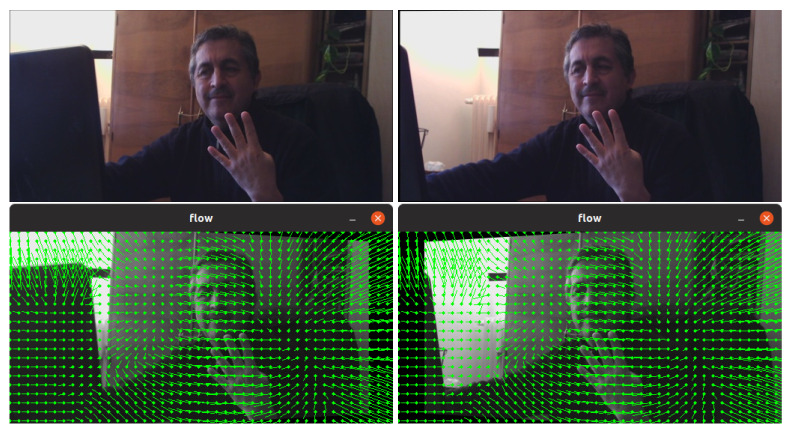
Left (L) and right (R) views (that will form 0–31 frames by morphing) of a stereoscopic image. The morphed displacements vectors of the optical flow data are also shown.

Our method for 3D multi-view then follows closely the algorithm of Baumann for animating stereograms [5]. We automatically apply optical flow deformations based on DeepFlow (which outputs *.flo* files) to our pairs of L–R images in order to morph between them. This alignment of the two (dissimilar) images, followed by a gradual fade out from one image to the other, can give an acceptable illusion of depth and (parallax) motion to the viewer (see examples in [5]). The optical flow, or vector field, describing movement between our stereoscopic images is shown in Figure 1.

In particular, this continuous interpolation by optical flow-based image warp enabled us to control the gradually cross-dissolving between pairs of stereo images. By fading out from one L image to the other R, we can then split a whole scene into a given number of middle component frames, as illustrated in Figure 1. In fact, optical flow computation is a key component in many computer vision systems designed for tasks, such as action detection or activity recognition, overcoming problems that arise in realistic videos, such as: motion discontinuities, occlusions, illumination changes, and ability to deal with (large) displacements.

We first generate *.flow* data for 32 different views (frames numbered 0–31 in the figure) starting from L to R images in an entirely unsupervised manner. This choice for the morphing process is done based on the nature of the Looking Glass HoloPlay [7].

**Figure 2:**
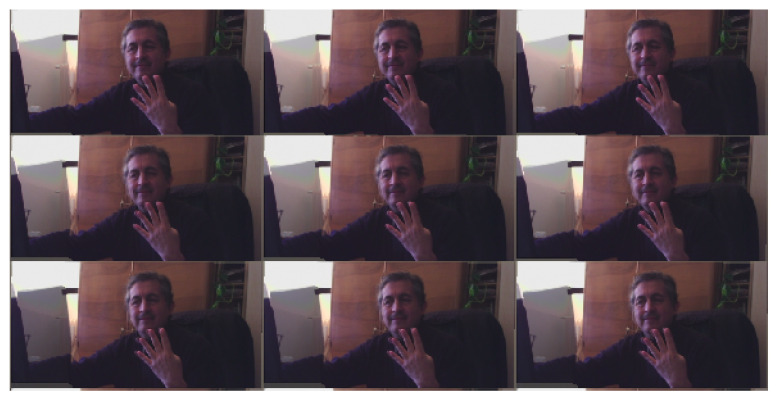
Central part of a 8 × 4 Quilt generated by morphing starting from the stereo images in Figure 1 via the *torch-warp* algorithm described in [5].

Next, the 32 different (*.png*) views from the morphing data are then converted to PNG24 to form the needed Quilt. We place the set of 32 view images (512 × 256p) in a single standard 4 × 8 Quilt image (2048 × 2048p), as in Figure 2, using *make_quilt.py* by SURFsara scripts [8]. The views start from the bottom left as the leftmost view (L-image) to the top right being the furthest right (R-image).

**Figure 3:**
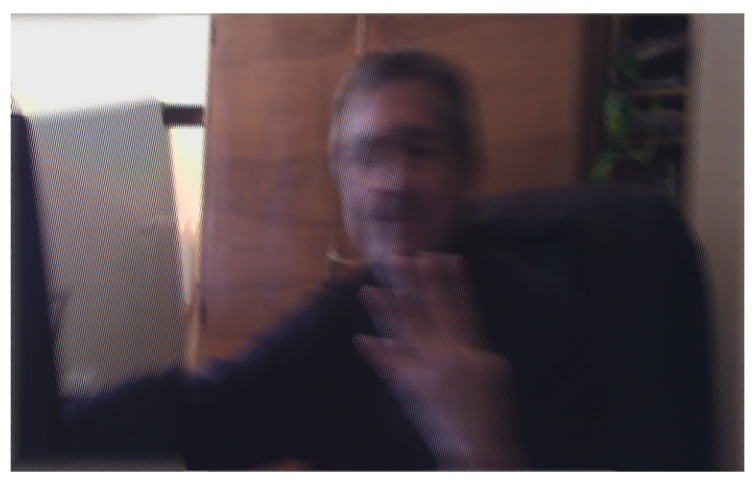
Native light-field image output of the Quilt in Figure 2 based on the per-device HoloPlay calibration *.json* values.

**Figure 4:**
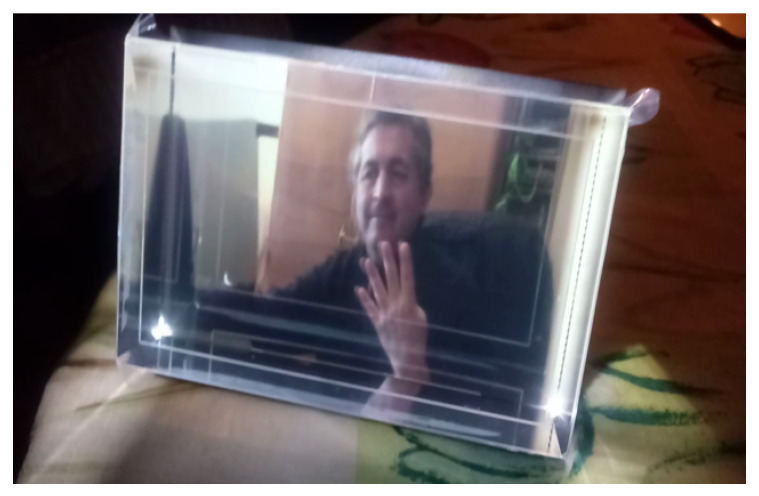
Multi-view hologram output from morphed stereoscopic images. See more in the video demo at: https://www.youtube.com/watch?v=6FAhmI-vtLQ.

Finally, we generate a Native image targeted to a specific HoloPlay device using the *quilt2native.py* script in [8], as shown in the example of Figure 3. We get the display calibration values (in the form of a standard data interchange file *.json*) from a Looking Glass Display using: *get_calibration_from_eeprom.py* from [8]. The multi-view 3D rendering of our morphed stereogram of Figure 3 can then be displayed on a HoloPlay device as appears in Figure 4. This output can also be seen in the video at: https://www.youtube.com/watch?v=6FAhmI-vtLQ.

In order to produce beautiful holograms, as in Figure 4, Looking Glass provides 32 (or 45 or more) discrete views or frames of a 3D scene, displaying these views over a ≈50${}^{\circ }$-wide view cone. This light-field arrangement tricks our visual perception into seeing 3D objects by parallax (i.e., moving the head around the scene, and by stereo vision (i.e., presenting different perspectives to each eye).

## 3. Discussion

Each Looking Glass holds its own calibration data for correct rendering and, inside its render volume, different depths have different optical properties [7]. The depth where things look sharpest is at the so-called Zero-Parallax Plane in the middle of the display (in our case, around frame 16). Objects in this plane show up in the same pixel-space position for all multi-views. Objects in the scene that are nearer or further than this plane undergo parallax. The Looking Glass HoloPlay provides a novel glasses-free way to preview 3D objects and scenes, as in Figure 4.

A generic expression for the relation between the pixels of a slanted lenticular 3D-LCDand the multiple perspective views was first derived by Cees van Berkel [12]. Each sub-pixel on the 3D-LCD is mapped to a certain view number and color value (i.e., in the light-field domain). If *i* and *j* denote the panel coordinates for each sub-pixel, then

(1) $$N_{i,j}=N_{tot}\left(i-i_{off}-3j\;tan\left(\alpha \right)\right)mod\left(P_{x}\right)/P\;\;\;,$$

where *N* denotes the view number of a certain viewpoint, α the slanted angle between the lenticular lens and the LCD panel, and $P_{x}$ is the lenticular pitch.

**Figure 5:**
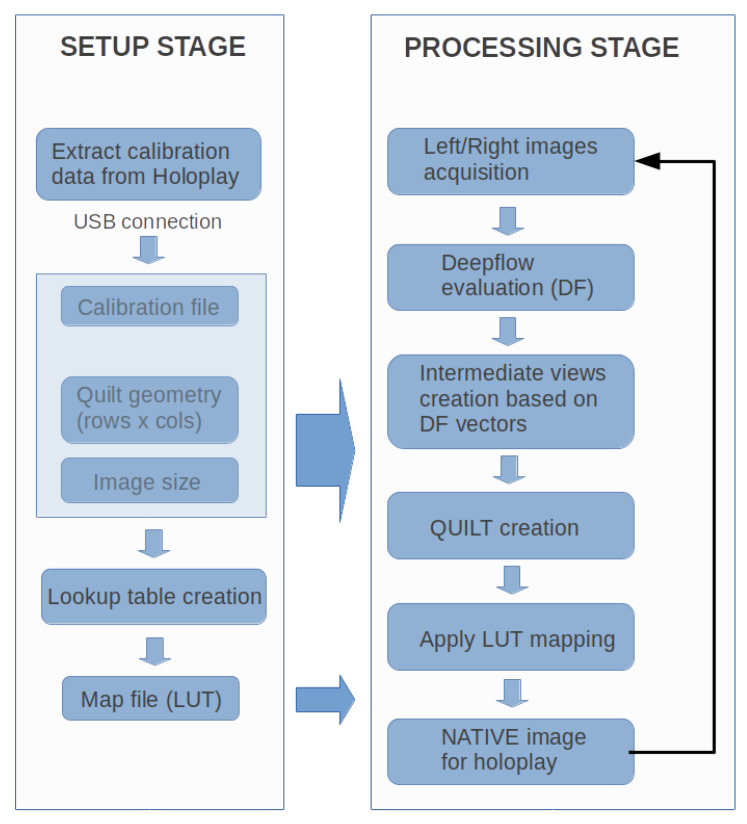
Flow diagram of the proposed algorithm starting from a stereoscopic image. A multi-view representation of a three-dimensional (3D) scene is obtained without using complex and expensive acquisitions via multiple cameras. LUT = Lookup Table.

**Figure 6:**
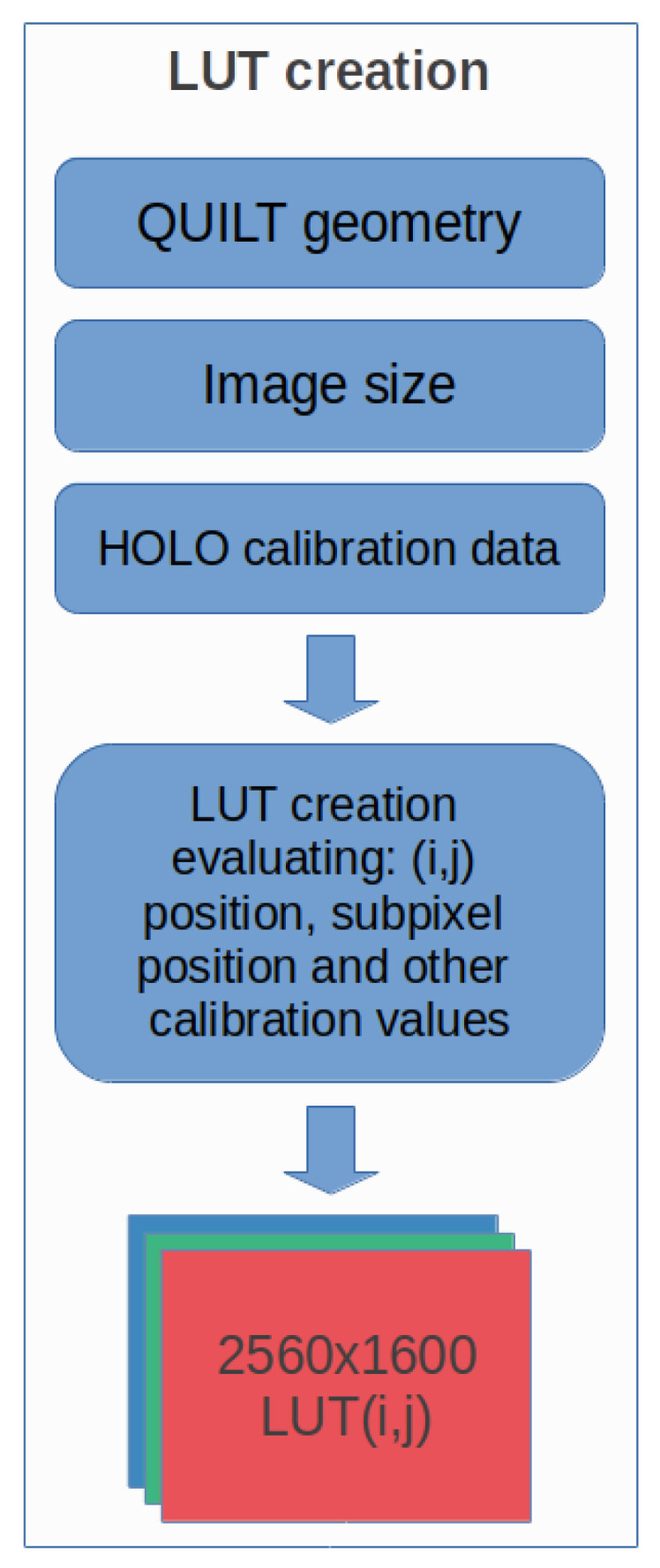
Basic steps of our LUT algorithm.

We created multi-view images via Equation (1) starting from a stereoscopic scene as illustrated in the flow diagram of in Figure 5. We put together ideas from optical flow techniques, morphing deformations, and light-field 3D rendering under Linux Ubuntu O.S. 2D morphing yield reasonable and better 3D visual results when it works correctly along the horizontal direction, as required by the optical elements adopted by the Looking Glass HoloPlay [7]. Upscaling multiple views (e.g., upscaling from the Quilt to the Native Looking Glass image) requires lots of CPU resources and increases system complexity [13,14,15]; however, with the generation of a device-dependent LUT table whose steps are described in Figure 6, this time can be considerably reduced. When the Quilt representation is created, we map each of those pixels in the destination Native image.

Fading from misaligned L to R images can cause the other intermediate parts of the whole scene to blur and get distorted. The limits of the morphing process in stereo animation, as compared with the use of depth map, is that morphing cannot provide complete information on distant regions. It mainly extrapolates the parallax encoded in the images by combining information from nearby pixels only. It is essentially a purely local method [16].

However, the application of 2D morphing to create the (32 or 45) required 2D views for the Looking Glass Quilt poses minimum geometric constraints on the reconstructed 3D scene via the alternate light-field projections. Morphing between the two images taken simultaneously with the ELP stereo webcam produces a nice illusion of 3D throughout the multi-views of a scene.

Optical flow techniques are relatively sensitive to the presence of occlusions, illumination changes, and out-of-plane movements. These factors lead to noise and to obtain translation motion discontinuities between the neighborhoods of two consecutive images. The key processing steps in the flow field [5,6] include the matching by polynomial interpolation to approximate pixel intensities in the neighborhood, warping and optimization without an explicit regularization. We have found that to generate *.flow* data for 32 views, optical flow techniques can lead to a reasonable accuracy to reconstruct 3D reality from stereoscopic images. The CPU time for creating these sets of view frames, and especially the final Looking Glass Native image, can take few seconds. This process still needs to be optimized.

As mentioned, in order to map the Quilt into the final Native image for a display on the holographic Looking Glass HoloPlay, it is necessary to apply the complex algorithm of Equation (1) that depends on input parameters of the physical structure of this device. Every HoloPlay monitor, in fact, possesses unique calibration parameters (such as pitch, slope, dpi) set in the phase of manufacturing. By making use of this calibration, and applying light-field geometric transformations, one then gets the multi-view image reproduced in the Looking Glass HoloPlay. This mapping procedure requires considerable calculation power, since the final Native image is at a resolution of 2560 × 1600p with three color channels—such that, once the pixel to be mapped is fixed, the map value for each color channel implies separated calculations. In essence, this procedure, as such, would become computationally expensive and difficult to apply in applications for a real-time video in 3D.

Since the mapping matrix depends on the geometric position of each pixel, and on the calibration parameters of the display, we constructed a LUT to replace run-time computation and save processing time.

This LUT is created only once at the beginning of the mapping process: Quilt → HoloPlay image, and then used for all the images that need to be visualized. We create the array as follows: First, we allocate three matrices for the three color channels RGB of size 256 × 1600p × 2. Each matrix provides the X coordinate of the Quilt from which we take the corresponding value and the Y coordinate. This explains the multiplication of the resolution 2560 × 1600p by 2. To avoid unnecessary waste of resources and consume the least possible amount of RAM memory, each element of the matrices is made of type *uint16_t* (the *uint8_t* type would allow to address maximum values of 255). Secondly, all the positions of the pixels 2560 × 1600p are scrolled, and we calculate the mapping value for each pixel on the Quilt image. Next, once the mapping value has been calculated, the value is stored in the three different allocated matrices.

Finally, once the map filling procedure has been completed, we save the three matrices in binary format. Then, at each successive time step, it is possible to reload the matrices (without the need of recalculating them) and apply the mapping automatically to all the necessary images. This procedure allows significant speeding up of the mapping procedure—the rendering operation of the final Native image is essentially achieved by accessing the elements of the three matrices to map the Quilt pixels on the final Native image for a dynamic display on the HoloPlay.

We implemented a new C-library MORPHOLO (see http://www.morpholo.it) that includes the LUT to quantify by bench-marking the timing to convert the Quilt into the Native image between the direct, classic method of Equation (1)—or, similarly to those of the SURFsara scripts [8]. Our first preliminary test for the statistics of the single files inside the C-library indicates that the total conversion time as is illustrated next.

**Figure 7:**
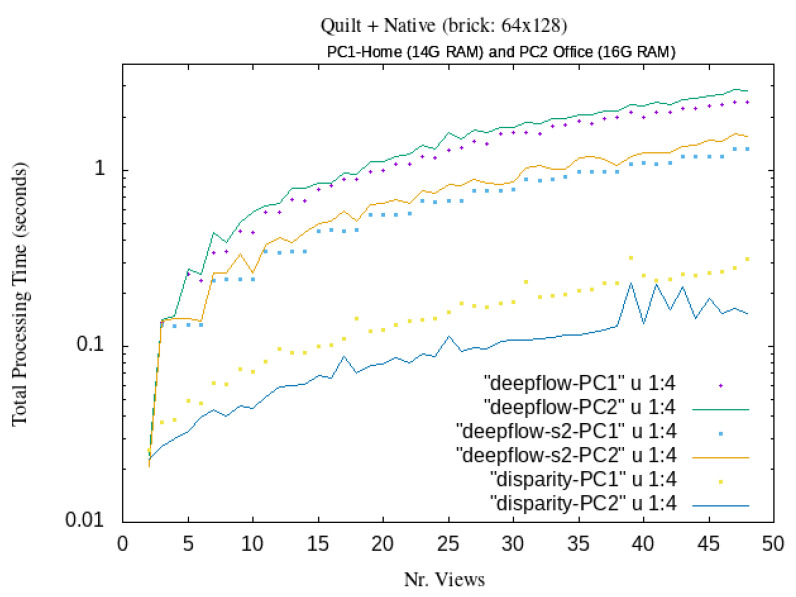
Computational timing of the MORPHOLO mapping procedure from the stereoscopic image to the Native output image on Linux Ubuntu O.S. for different number of multi-views.

The estimate of the total processing time employed for the generation of MORPHOLO holograms starting from a set of small-resolution stereoscopic images is shown in Figure 7. As quantified in these curves, total processing time steps below 0.1 s, in between 10-*to*-35 different views, can be obtained with the use of the present LUT. This implies that in 1 second, we can generate ten or more different Native frames, opening a gateway to achieve 3D real-time video streaming with DeepFlow and Disparity map morphing algorithm.

This means that the implementation of LUT allows reduction in the computing time of about 50%. One could even reduce the computation time by a factor of 4, by allocating the four threads of a Raspberry Pi 3 with four processors to the Quilt image of Figure 2 divided in four parts (with successive eight views each). These possibilities open the path for producing multi-view 3D streaming in real-time with a simpler and faster algorithm and shooting stereo images.

## 4. Conclusions

We have proposed a rapid conversion approach that allows to transform stereoscopic images into holograms via the automatic implementation of a LUT and unsupervised morphing deformations between the L and R stereo images. The key idea here is just to start from the pair of structured stereoscopic 2D images with no additional information about any in-between view to create the holograms. Under this method, we establish correlations between 2D image observations and their 3D display on a Looking Glass HoloPlay monitor.

In order to reduce the evaluation of the Equation (1) for every pixel, we have implemented a LUT, which maps each pixel of the native image to the right pixel in the Quilt representation. Our new LUT approach highly reduces the computational cost of the mapping procedure. This LUT can be created before starting the complete algorithm, and it can be applied for every new created Quilt (while avoiding the whole Equation (1) evaluation). Therefore, our contribution here to the state-of-the-art includes the LUT generation, an actual evaluation metrics of systematic tests via the use of our original LUT for the Quilt and Native for future live 3D video streaming, and the use a generic algorithm capable to get images from a stereo camera. The diagram of the algorithm to produce multi-view as fast as possible and reasonably is synthetized and illustrated in Figure 5, Figure 6 and Figure 7.

Future work could include the potential application of this method in the field of live 3D streaming using cost effective hardware and no headsets. In principle, it would be also possible to interact with such real-time holograms displayed in the HoloPlay screen with a Leap Motion controller [7].

Ultimately, the different set of techniques discussed in this work still needs to be optimized and further experimented when combined all together. Work along these lines is under development.
